# Therapeutic Potential of Bioactive Compounds in Edible Mushroom-Derived Extracellular Vesicles: Isolation and Characterization of EVs from *Pleurotus eryngii*

**DOI:** 10.3390/ph18091362

**Published:** 2025-09-12

**Authors:** Gaia Cusumano, Agnese Bertoldi, Eleonora Calzoni, Husam B. R. Alabed, Roberto Maria Pellegrino, Lorena Urbanelli, Gokhan Zengin, Giancarlo Angeles Flores, Roberto Venanzoni, Paola Angelini, Carla Emiliani

**Affiliations:** 1 Department of Chemistry, Biology and Biotechnology, University of Perugia, 06100 Perugia, Italy; gaia.cusumano@dottorandi.unipg.it (G.C.); agnese.bertoldi@dottorandi.unipg.it (A.B.); husambr.alabed@unipg.it (H.B.R.A.); roberto.pellegrino@unipg.it (R.M.P.); lorena.urbanelli@unipg.it (L.U.); giancarlo.angelesflores@unipg.it (G.A.F.); roberto.venanzoni@unipg.it (R.V.); paola.angelini@unipg.it (P.A.); carla.emiliani@unipg.it (C.E.); 2 Centro di Digitalizzazione del Patrimonio Culturale (CeDiPa), University of Perugia, 06100 Perugia, Italy; 3 Department of Biology, Science Faculty, Selcuk University, 42130 Konya, Turkey; gokhanzengin@selcuk.edu.tr; 4 Centro di Eccellenza Materiali Innovativi Nanostrutturati (CEMIN), University of Perugia, Via del Giochetto, 06123 Perugia, Italy

**Keywords:** extracellular vesicles, *Pleurotus eryngii*, mycelium-derived EVs, mushroom-derived EVs, antioxidant activity, metabolomics

## Abstract

**Background/Objectives**: Over the past twenty years, there has been a rapid increase in studies aimed at comprehending how cells communicate with each other via Extracellular Vesicles (EVs), accompanied by a heightened interest in plant-derived extracellular vesicles due to their potential relevance in dietary supplementation and therapeutic applications. However, there is a limited amount of research on extracellular vesicles derived from mushrooms (MDEVs). Among edible mushrooms, *Pleurotus eryngii* is peculiar due to its flavor and interesting nutritional profiling. It also produces a wide array of secondary metabolites including biologically active compounds with many health-promoting benefits such as anticancer, antioxidant, antitumor, antiviral, antibacterial, antidiabetic, and anti-hypercholesteremic activities. The aim of this work has been to isolate EVs from the fruiting body and mycelium of *P. eryngii* in order to investigate their potential applications as nutraceuticals. **Methods**: MDEVs were isolated by differential and density gradient centrifugation, characterized by Nanoparticle Tracking Analysis (NTA), Scanning Electron Microscopy (SEM) and immunoblotting, and subjected to metabolomic and phenolic profiling. Their antioxidant potential was assessed through in vitro radical scavenging (DPPH, ABTS) and metal-reducing (CUPRAC, FRAP) assays. **Results**: The findings suggest that mycelium-derived EVs may represent a valuable source of high-quality MDEVs, which exhibited promising antioxidant properties in all assays conducted, particularly in radical scavenging assays. **Conclusions**: These results highlight the potential of *P. eryngii* mycelium-derived EVs as a novel natural source of bioactive compounds, paving the way for future applications in nutraceutical and therapeutic fields.

## 1. Introduction

Extracellular Vesicles (EVs) are bilayer-lipid nanoparticles released by all living organisms, which play essential roles in intercellular communication, molecular transport, and immune modulation [1,2]. While the investigation of EVs is well-established in mammals and bacteria, research on EVs in fungi and plants is still under-explored. Moreover, in fungal research, the terminology and classification of EVs remain ambiguous, as no specific markers are available to clearly distinguish subtypes such as exosomes or microvesicles. This taxonomic uncertainty emphasizes the novelty of investigating mushroom-derived EVs, where standardized definitions and markers are still lacking. Recent discoveries have pointed out their biochemical composition, functional activities, and biotechnological possibilities [3,4]. Edible mushrooms are garnering significant attention due to their nutritional qualities and pharmacological benefits, which are linked to the presence of active compounds such as polysaccharides, polyphenols, sterols and bioactive proteins. These molecules have been associated with multiple biological effects, including immunomodulatory, anti-inflammatory, anticancer, antioxidant, and anti-diabetic activities, thus supporting the role of mushrooms as both functional foods and sources of nutraceuticals [5]. Among the most cultivated edible mushrooms, *Lentinula edodes* (Shiitake) has also been widely studied as a functional food. Several bioactive constituents have been isolated from this source, including polysaccharides, flavonoids, sterols and proteins [6,7]. Recently, Yang et al. isolated exosomes from the fruiting body of *L. edodes*, highlighting their ability to inhibit, in vitro, the growth of CaCo2 tumor cells [6].

In light of this scientific evidence, the purification of EVs from edible mushrooms with medicinal properties appears to be a particularly captivating field of research. Within edible mushrooms, the genus *Pleurotus* (Fr.) P. Kumm. (Pleurotaceae, Basidiomycota) is also known for its numerous species with culinary and medicinal importance [8,9,10]. This genus primarily consists of white-rot saprotrophic species that thrive on decaying wood and plant residues. The ability of *Pleurotus* spp. to fruit across a wide range of temperatures and efficiently utilize lignocellulosic substrates makes it an attractive candidate for large-scale mushroom cultivation worldwide [11]. Among *Pleurotus* species, *Pleurotus eryngii* (DC.) Quél. is considered the foremost species in its genus due to its superior cap and stem quality, extended shelf life, and rich content of bioactive compounds such as carbohydrates, peptides, and dietary fiber [12]. Additionally, this species is noted for its high vitamin content [13,14]. Different in vitro studies have highlighted its health benefits, including antioxidant, antimicrobial, anti-inflammatory, anticancer, and immunomodulatory effects [15,16,17,18]. These therapeutic activities are associated with specific molecular mechanisms. In particular, polysaccharides and phenolic compounds from *P. eryngii* have been reported to modulate the Nrf2/ARE antioxidant pathway, enhancing cellular defenses [19]. Moreover, extracts rich in polyphenols can suppress NF-κB activation, thereby reducing pro-inflammatory responses [20]. In addition, bioactive metabolites have been shown to induce apoptosis in tumor cells through mitochondrial and caspase-dependent pathways [19]. Furthermore, immunomodulatory effects are mainly linked to the activation of macrophages and the stimulation of cytokine production, which together contribute to strengthening the immune response [16].

It has already been demonstrated that EVs from non-animal sources, including plants and filamentous fungi, can transport a lot of bioactive molecules such as proteins, lipids, nucleic acids, and secondary metabolites which can exert peculiar biological activity. Therefore, it is plausible to hypothesize that macrofungi may also exhibit this capability [3,21,22,23,24].

Hence, there is an increasing interest in purifying EVs from these sources, to assess whether these lipid nanocarriers are able to transport the same bio-active molecules found in cellular extracts and mediate interspecies cross-talk mechanisms. Recent proteomic and metabolomic studies have revealed that MDEVs from fungi of the genus *Cryptococcus, Candida, Saccharomyces* and others may carry not only small RNAs and polysaccharides but also bioactive lipids, which are involved in modulating immune responses and cellular signaling pathways [25]. Additionally, EVs derived from *Morchella sextelata* and *Phellinus linteus* have shown interesting therapeutic properties, exhibiting antimicrobial, immunomodulatory and anticancer activities, making them attractive candidates for drug delivery [26,27]. Their nanoscale size (typically ranging from 30 to 200 nm) allows them to efficiently penetrate biological membranes, which may contribute to their ability to penetrate physiological barriers, including the blood–brain barrier (BBB) [28]. Furthermore, their natural origin and biocompatibility highlight their potential as novel nanocarriers in pharmaceutical applications. EVs derived from edible fungi are naturally present in their tissues and, consequently, can be introduced into the human body through the diet. Recent studies on Plant-derived Extracellular Vesicles (PDEVs) have shown that these EVs exhibit high stability in the gastrointestinal tract and possess specific tissue-targeting capabilities, protecting the encapsulated biomolecules from degradation, and MDEVs possibly share these properties [29,30]. Despite these promising characteristics, research on MDEVs is still in its infancy, with many fundamental questions regarding their biogenesis, molecular cargo and mechanisms of action still unresolved. The molecular processes that control the formation and secretion of MDEVs are not well comprehended. Current findings indicate that MDEVs might form in endosomal compartments, much like mammalian exosomes, or be discharged through budding at the plasma membrane [31]. In this context, the aim of this work was to isolate EVs from both the fruiting body and mycelium of the edible mushroom *P. eryngii* to characterize these EVs and gain insight their potential as a source of metabolites with relevant biological properties mirroring those of *P. eryngii* extract. In particular, the use of a pure culture of mycelium from *P. eryngii* was evaluated in order to standardize the production process of MDEVs. Moreover, EVs were isolated through differential ultracentrifugation at 40,000× *g* and 100,000× *g*, conditions that enrich vesicles of different size ranges. Comparing these subpopulations is important to highlight possible differences in biochemical composition and biological functions. With these assumptions, an isolation protocol from the fruiting body and mycelium of *P. eryngii* was set up and MDEVs were analyzed based on their morphology, metabolic content, and antioxidant properties, assessed through DPPH and ABTS in vitro assays for radical scavenging activity, and FRAP and CUPRAC in vitro assays for metal-reducing antioxidant power.

## 2. Results

### 2.1. Characterization of Mushroom-Derived Extracellular Vesicles

#### Scanning Electron Microscopy (SEM), Nanoparticle Tracking Analysis (NTA) and Immunoblotting Analysis

EVs were purified by differential ultracentrifugation, as described in Section 4.2.

**Figure 1:**
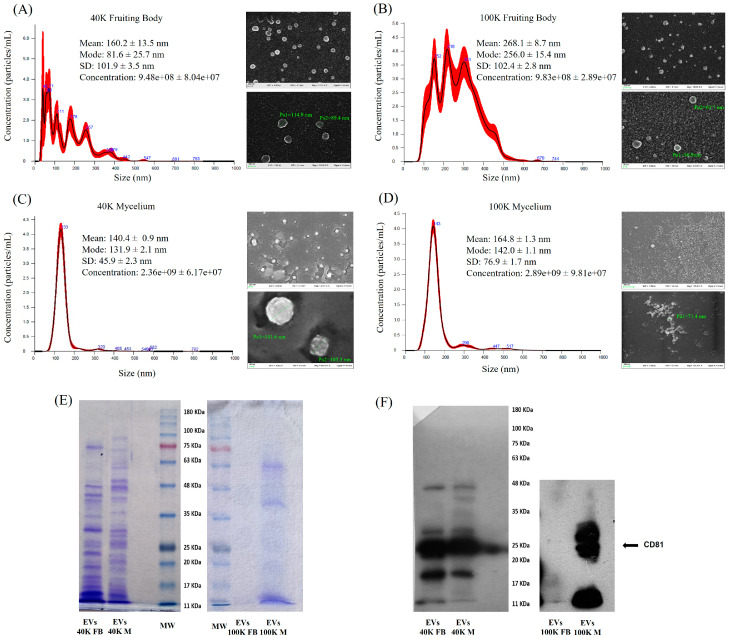
Characterization of *P. eryngii*-derived extracellular vesicles. (**A**–**D**) NTA and SEM analyses of 40 K and 100 K EV fractions from fruiting body (**A**,**B**) and mycelium (**C**,**D**). (**E**) Coomassie-stained SDS-PAGE of 40 K and 100 K EVs from fruiting body (FB) and mycelium (M). (**F**) Immunoblotting for CD81 in EV fractions.

Both fractions of MDEVs isolated from the fruiting body and mycelium of *P. eryngii* were analyzed by Nanoparticle Tracking Analysis (NTA) and Scanning Electron Microscopy (SEM) to assess size distribution, purity, and morphology (Figure 1A–D).

For EVs derived from the fruiting body, NTA measurements revealed a high degree of heterogeneity and poor resolution of particle size, likely due to the presence of contaminating debris, which hampered accurate quantification (Figure 1A,B). On the other hand, SEM analysis provided clearer morphological information: 40 K fraction EVs appeared as spherical structures with an estimated diameter of 80–100 nm, while those from the 100 K fraction were smaller, averaging around 60 nm. However, the background of 100 K samples displayed a dense matrix of smaller particles, suggesting residual contamination or vesicle fragmentation.

Conversely, EVs isolated from the mycelium showed cleaner profiles. NTA of both 40 K and 100 K fractions yielded well-defined particle distributions, with average sizes of approximately 130–140 nm (Figure 1C,D). SEM confirmed the presence of vesicular structures in the 40 K fraction, though particles exhibited irregular shapes. Also in this case, 100 K mycelial EVs were more difficult to resolve individually under SEM, consistent with their lower size range.

These observations highlight differences in EV purity and morphology depending on the biological source and isolation protocol, with mycelium-derived EVs showing higher quality and homogeneity compared to those from the fruiting body.

To further discriminate the characteristic of *P. eryngii* MDEVs from different sources, Coomassie Brilliant Blue G-250 staining was also performed to compare the protein content of *P. eryngii* EVs. SDS-PAGE gels were loaded with equal amounts of protein (10 μg) from the 40 K and 100 K fractions of both fruiting body (FB) and mycelium (M) (Figure 1E). The 40 K fractions exhibited more intense and heterogeneous protein bands compared to the 100 K counterparts, indicating a higher overall protein content. Notably, the 100 K fraction from the fruiting body showed a markedly poor protein profile, with faint or undetectable bands, whereas the 100 K fraction from the mycelium retained a more complex and detectable pattern. These qualitative findings were corroborated by quantitative analysis. Considering a starting biomass of 2.5 g, the total protein yield in the 40 K fractions was 2.7 mg (FB) and 1.5 mg (M), while the 100 K fractions yielded 2.0 mg (FB) and 1.2 mg (M). These findings highlight both a greater protein yield and a more complex composition in the 40 K fractions.

To confirm the vesicular nature of the isolated particles, immunoblotting was performed for CD81, a well-established tetraspanin marker commonly enriched on the surface of small extracellular vesicles, including exosomes, and widely used as a hallmark of animal vesicular identity and purity. To date, no specific antibodies for MDEVs have been identified; therefore, commonly used markers for animal-derived EVs have been employed [26]. In this study, only CD81 marker produced a positive signal. Although CD81 is a standard marker for animal EVs, it has been used in fungal EV studies as a comparative marker due to the identification of putative homologous proteins, allowing a preliminary assessment of vesicular identity while acknowledging potential evolutionary divergence. As shown in Figure 1F, CD81 was detected in the 40 K fractions from both the fruiting body and mycelium (40 K FB and 40 K M), as well as in the 100 K fraction from the mycelium (100 K M). The absence of detectable CD81 in the 100 K fruiting body fraction is consistent with the weak protein profile observed in the corresponding Coomassie-stained gel, further supporting the higher-level vesicle yield and quality of mycelium-derived preparations.

**Figure 2:**
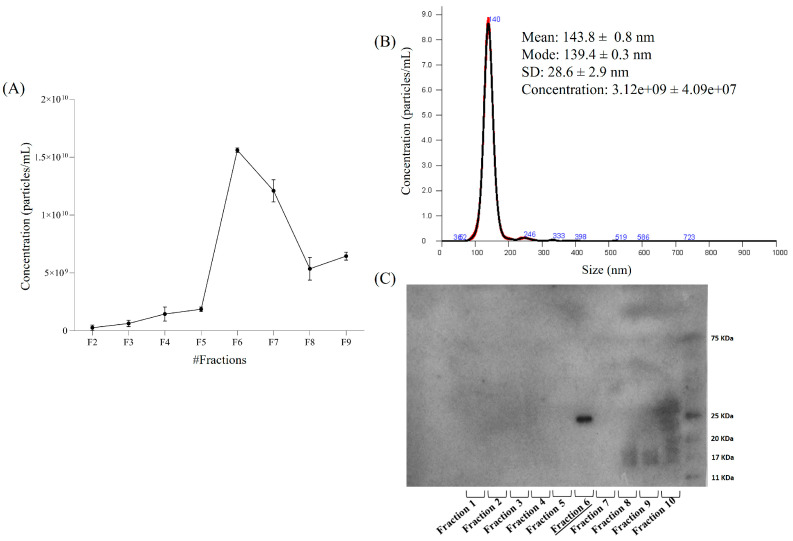
Analysis of 40 K mycelium-derived EVs after density gradient separation. (**A**) Particle concentration of different density gradient fractions is reported as Particles/mL obtained by NTA; (**B**) Size distribution of the most enriched fraction 6 analyzed through NTA and (**C**) CD81 detection in gradient fractions (from 1 to 10) obtained by immunoblotting.

Considering the superior purity observed in the 40 K mycelial EV fraction, this sample was subjected to further purification by density gradient ultracentrifugation on iodixanol gradient. Ten gradient fractions were collected and analyzed by NTA to quantify particle concentrations (Figure 2A). Among these, fraction 6 exhibited the highest vesicle concentration and displayed a clean, monodisperse profile centered at approximately 140 nm (Figure 2B), confirming the enrichment of vesicles within the expected size range. To further validate the enrichment of vesicles following density gradient ultracentrifugation, immunoblotting for CD81 was also performed on the ten fractions collected from the 40 K mycelial EV sample. Among them, only fraction 6 showed a positive signal for CD81 (Figure 2C), corroborating the results obtained by NTA in terms of both particle concentration and size distribution. The results confirm that EVs of mycelial origin have a high degree of homogeneity. Furthermore, purification by density gradient allows obtaining a clean, homogeneous and enriched fraction of MDEVs.

### 2.2. Untargeted LC-MS/MS-Based Metabolomics Analysis of MDEVs

**Figure 3:**
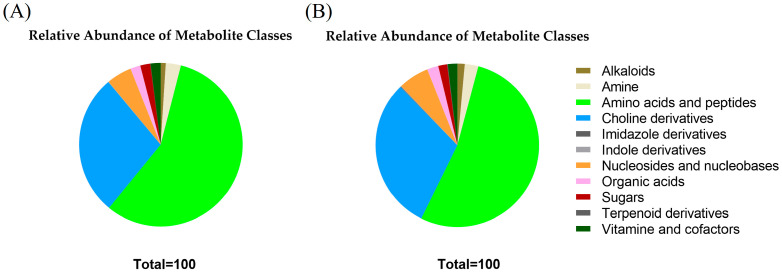
Relative abundance of metabolite classes detected in the 40 K fraction (**A**) and 100 K fraction (**B**) of *P. eryngii* MDEVs.

To gain insight into the MDEVs molecular cargo, untargeted LC-MS/MS metabolomics analysis was also performed on *P. eryngii* mycelium-derived EVs. The results revealed a complex metabolic composition in the 40 K and 100 K fraction of *P. eryngii* mycelium EVs, with a broad range of polar metabolites identified (Figure 3A,B and Table S1), including amino acids (e.g., L-leucine, L-phenylalanine, L-lysine, L-valine), dipeptides (e.g., γ-L-glutamyl-L-alanine, N-L-γ-glutamyl-L-leucine), nucleosides (adenosine, guanosine), and choline derivatives such as betaine and glycerophosphocholine. Several alkaloids (harmane, norharmane), indole and imidazole derivatives, organic acids (e.g., succinic and citric acid), sugars, and cofactors (e.g., pantothenic acid, nicotinic acid) were also detected, indicating a variegated biochemical content potentially involved in redox regulation and cell signaling.

Among free amino acids, small peptides, and modified peptides revealed by metabolomic analysis, it was observed the presence within MDEVs of γ-aminobutyric acid (GABA), L-glutamate, L-arginine, cyclo(isoleucylprolyl), and pyroglutamyl derivatives (Table S1).

Moreover, some polyphenolic compounds were also identified and quantified. In Table 1 the compounds found in the 40 K fraction are reported and their amounts compared with whole mycelium extract. These included (−)-epicatechin, epicatechin gallate, caffeic acid, rutin, and salicylic acid. Similar polyphenolic profiles were observed in the 100 K fraction, as the percentages were substantially overlapping with those found in the 40 K fraction.

EVs derived from *P. eryngii* mycelium were found to be enriched in interesting polyphenolic species compared to the corresponding extract, such as caffeic acid, (−)-epicatechin, rutin, gallic acid and trans-4-coumaric acid. It is widely recognized that these metabolites play a significant role in anti-inflammatory and antioxidant activities [32,33,34,35,36]. Comparison with the mycelial extract, which in this case represents the polyphenolic component of the *P. eryngii* metabolome, demonstrated that certain metabolites ((−)-epicatechin, rutin, and trans-4-Coumaric acid) are more enriched in EVs, which therefore represent an interesting cargo of these MDEVs.

These results imply that 40 K MDEVs contain fascinating bioactive phytochemicals that may exhibit noteworthy biological activities.

**Figure 4:**
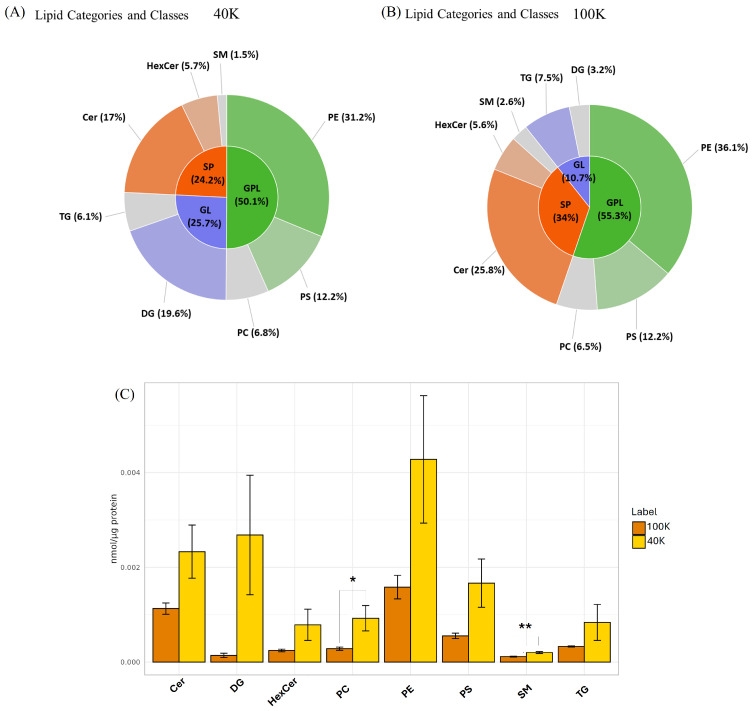
Relative abundance of major lipid categories and their corresponding lipid classes found in 40 K (**A**) and 100 K (**B**) fractions of *P. eryngii*. Comparison of lipid class content in 40 K and 100 K MDEVs, (concentration expressed in nmol/μg protein) (**C**). Data are expressed as mean ± SD (*n* = 3). Statistical analysis was performed using *t*-test. Asterisks indicate significant differences between 40 K and 100 K fractions: * *p* < 0.05, ** *p* < 0.01.

Lipidomic analysis was also performed to characterize the non-polar components associated with the membrane of MDEVs. Initially, an appropriate extraction of the lipid component was performed as reported in the materials and methods section using the MMC method [37]. The results, shown in Figure 4, further confirm the lipidic nature of these vesicles, with several representative lipid classes detected including ceramides (Cer), phosphatidylcholines (PC), phosphatidylethanolamines (PE), phosphatidylserines (PS), diacylglycerols (DG), triacylglycerols (TG), and sphingomyelins (SM). These lipid classes were grouped into three major lipid categories: glycerophospholipids (GPL), sphingolipids (SP), and glycerolipids (GL).

Noteworthy, the 40 K fraction exhibited a characteristic lipid composition compared to those of the 100 K fraction. In 40 K MDEVs, glycerolipids (GL) represented a substantial proportion of the total lipid signal (~25.7%), indicating a peculiar enrichment in neutral lipids, particularly DG (~19.6%) and TG (~6.1%). Despite the overall dominance of glycerophospholipids (GPL) in both populations, 40 K MDEVs showed a lower relative GPL content (~50.1%) compared to 100 K (~55.3%), yet a more balanced distribution among key phospholipid classes. Specifically, PE (31.2%), PS (12.2%), and PC (6.8%) were all well represented. Furthermore, the sphingolipid (SP) fraction was higher in 40 K fraction (~24.2%), mainly due to the presence of ceramides (~17%) and hexosylceramides (~5.7%). The comparison between lipid classes of the two fractions is further detailed in Figure 4C. When normalized to total protein content (nmol/μg protein), 40 K MDEVs were found to contain a higher relative abundance across all lipid classes. This enrichment was particularly evident for phosphatidylcholines (PC) and sphingomyelins (SM), which showed statistically significant increases (*p* < 0.05) in the 40 K fraction. This observation is in line with comparative lipidomic analyses showing that large extracellular vesicles contain higher proportions of plasma-membrane–associated lipids, such as phospholipids and sphingolipids, compared with smaller vesicles [38]. Moreover, a recent study on shiitake-derived nanovesicles confirmed a strong enrichment in phospholipids, supporting the notion that fungal EVs are particularly enriched in glycerophospholipid classes [6]. Further differences at the level of individual lipid species within each class were also detected, as detailed in Figure S1, highlighting specific molecular variants that contribute to the compositional divergence between the two vesicle populations. In addition to confirming the presence of a plethora of polar metabolites and phenolic compounds, the LC/MS untargeted analyses revealed compositional differences that parallel those observed in protein profiles. The 40 K fraction, in particular, showed enrichment in several polyphenols and in neutral lipids such as diacylglycerols and triacylglycerols, suggesting a vesicle population specialized in carrying antioxidants and signaling molecules. Conversely, 100 K vesicles displayed a higher proportion of glycerophospholipids and a more balanced distribution among key phospholipid classes, consistent with their smaller size. When considered together, the metabolomic and lipidomic data indicate that MDEVs are not uniform carriers but encompass subpopulations with specific biochemical signatures.

### 2.3. Antioxidant Activity of Mushroom-Derived Extracellular Vesicles

**Figure 5:**
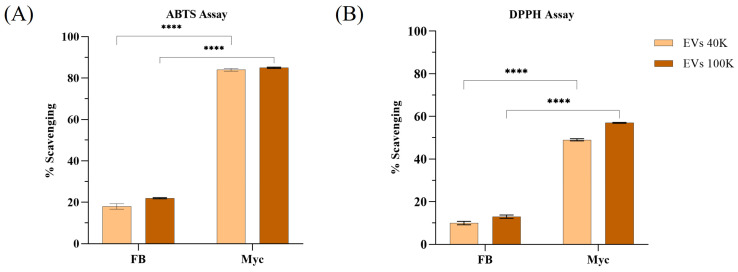
Percentage of radical scavenging by MDEVs assessed using (**A**) ABTS and (**B**) DPPH assays. Comparison between EV fractions isolated from fruiting body (FB) and mycelium (Myc). Data are expressed as mean ± SD (*n* = 3). Statistical analysis was performed using the *t*-test. **** *p* < 0.0001.

In order to investigate potential biological activities of EVs from *P. eryngii*, the antioxidant potential was evaluated using DPPH, ABTS, FRAP, and CUPRAC assays. As shown in Figure 5, MDEVs from mycelium exhibited substantial free radical scavenging activity in both DPPH and ABTS assays, with comparable performance between the 40 K and 100 K fractions. Otherwise, MDEVs isolated from fruiting body showed lower scavenging power, especially in the 40 K fraction.

Detailed quantitative results are reported in Table 2.

Mycelium MDEVs demonstrated greater antioxidant capacity across all assays. The 40 K mycelial fraction showed the highest values in DPPH (56.27 ± 0.48 µg TE/µg EVs), ABTS (89.61 ± 0.32 µg TE/µg EVs), FRAP (113.34 ± 5.04 µg TE/µg EVs), and CUPRAC (196.5 ± 2.76 µg TE/µg EVs) respected to FB 40 K counterpart. The 100 K mycelial fraction also exhibited relevant antioxidant capacity, particularly in the ABTS assay (117.15 ± 0.66 µg TE/µg EVs), while CUPRAC and FRAP values resulted lower than 40 K mycelial fraction. EVs derived from the fruiting body presented lower activity across all assays, with the 100 K fraction generally less active than the 40 K. In general, mycelium-derived 40 K EVs showed a better capacity to reduce metal ions than their fruiting body-derived counterparts, as well as a better free radical scavenging capacity. These data support that MDEVs, especially the 40 K mycelial fraction, demonstrate significant antioxidant capabilities and are the leading candidates for further investigation in terms of antioxidant and anti-inflammatory in biological environment.

## 3. Discussion

Extracellular Vesicles (EVs) originating from macrofungi have drawn more interest in recent years, given their potential to act as carriers of bioactive compounds that play significant roles in cellular signaling, defense mechanisms, and stress resilience [22,39]. However, despite these promising perspectives, several challenges remain before MDEVs can be exploited in applied settings. Current ultracentrifugation or size-exclusion methods, for example, are often time-consuming, and not suited for large-scale production, which hampers translational applications. Moreover, the heterogeneity of EV populations makes it difficult to obtain standardized preparations with reproducible biochemical profiles. In this contest, using mycelial-derived EVs could represent an innovative starting material source, because can be produced under controlled conditions in a defined environment [40]. In addition, while metabolomic and lipidomic analyses suggest that MDEVs are enriched in bioactive molecules with potential antioxidant and anti-inflammatory activities, their stability, biodistribution, and bioavailability in living organisms remain unexplored. In vivo studies are required to confirm whether the protective activities inferred from their molecular cargo can be translated into physiological effects. Addressing these issues will be crucial to advance the field and evaluate the biomedical potential of mushroom-derived EVs. Nonetheless, this area of study is still unripe relative to research on EVs from plants or mammals. The detailed characterization of their molecular content, particularly from edible or medicinal species, is currently lacking. Although extracellular vesicles are complex mixtures of biomolecules and it is difficult to attribute specific biological effects to individual components, evaluating their activity as complete phytocomplex systems is meaningful, since extracellular vesicles naturally act as carriers that release multiple bioactive molecules. This study presents an in-depth characterization of extracellular vesicles isolated from the fruiting body and mycelium of *Pleurotus eryngii*, with a focus on two distinct ultracentrifugation-derived fractions: 40 K and 100 K. Initial screening based on morphology, protein content, and purity highlighted substantial differences between these fractions and sources. Notably, the 40 K mycelial EVs consistently outperformed other isolates in terms of yield, structural integrity, and functional potential.

Morphological analysis by SEM and NTA revealed that fruiting body-derived EVs were often heterogeneous and contaminated with debris, particularly in the 100 K fraction, complicating downstream analyses. Conversely, the 40 K mycelial EVs displayed a cleaner, more homogeneous profile. These findings were corroborated by density gradient ultracentrifugation, which further refined the 40 K isolate and allowed for the identification of a highly enriched vesicle population in fraction 6, characterized by well-defined morphology and a consistent size distribution around 140 nm.

Untargeted LC-MS/MS profiling of the 40 K and 100 K mycelial EVs revealed a complex array of small molecules. The polar fraction was enriched with low-molecular-weight metabolites, including amino acids, nucleosides, dipeptides, alkaloids, organic acids, and intermediates of primary metabolism, which are often implicated in cellular signaling and stress responses. These findings align with previous studies indicating that fungal EVs can carry a variety of small metabolites involved in cell-to-cell communication and environmental adaptation [40,41]. The detection of neurotransmitter-related compounds such as GABA and glutamate may suggest a potential role in signaling pathways, while the presence of essential amino acids (e.g., leucine, isoleucine, lysine) points to a possible nutraceutical or metabolic function [35,36]. Furthermore, the identification of bioactive polyphenols, some of which are known to exert antioxidant or immunomodulatory effects, indicates that MDEVs may actively contribute to biological regulation in recipient cells [41,42]. (−)-epicatechin was detected at the highest percentage in MDEVs. This molecule has demonstrated efficacy in suppressing inflammation via inhibition of the NLRP3 inflammasome and modulation of the NF-κB signaling cascade, with therapeutic relevance in inflammatory pathologies such as gouty arthritis [33]. Similarly, Caffeic acid, another abundant molecule, exhibits strong antioxidant properties and exerts immunomodulatory and anti-inflammatory effects by inhibiting NF-κB pathway activation, thus reducing pro-inflammatory cytokine production such as IL-6 and TNF-α [36]. Gallic acid has been widely studied for its potent antioxidant capacity and ability to modulate oxidative stress and inflammatory processes [35]. Moreover, trans-4-coumaric acid has shown promising metabolic and anti-inflammatory activity, partly mediated by activation of brown adipose tissue and modulation of systemic energy metabolism [34]. The presence of these molecules in MDEVs suggests that these nanostructures may play a functional role beyond cargo transport, potentially participating in redox regulation and immune modulation in recipient systems. Although the enrichment of bioactive polyphenols such as (−)-epicatechin and caffeic acid in MDEVs suggests potential modulation roles, it is important to consider their stability in vivo. Many polyphenols exhibit limited bioavailability due to degradation under physiological conditions (e.g., pH, enzymatic activity), as well as poor solubility and permeability [43]. For these reasons, encapsulation within extracellular vesicles may confer enhanced protection and potentially improve stability and targeted delivery; for example, curcumin encapsulated into exosomes has been shown to be significantly more stable compared to the free molecules [44]. Thus, MDEVs could play a critical role in safeguarding polyphenols from degradation and enhancing their functional delivery to recipient cells.

The apolar fraction contained vesicle-associated lipids such as ceramides, phosphatidylcholines, and triglycerides and other representative classes, confirming the structural integrity and complexity of these vesicles. The overall enrichment in sphingolipids, particularly in the 40 K fraction, reflects a distinctive membrane architecture, potentially linked to enhanced vesicle stability, and lipid-mediated signaling. In fact, lipids not only maintain membrane integrity and influence vesicle formation but also participate in biological activities such as immune modulation and oxidative stress regulation [42,43]. In particular, lipid composition is known to influence vesicle–cell interactions, including uptake mechanisms. For example, exosome-associated ceramides and phosphatidylserines have been shown to modulate endocytosis and membrane fusion, thereby affecting the efficiency of cargo delivery [45]. Thus, the distinctive lipid signature of *P. eryngii*-derived EVs, particularly their sphingolipid enrichment, may play a role not only in structural stabilization but also in determining cellular tropism and uptake efficiency. The integration of lipid and polar metabolite content may enable MDEVs to provide a coordinated biological effect, where membrane stability, signaling, and bioactive molecule delivery work together in target cells.

Moreover, antioxidant assays showed that mycelial MDEVs exhibit considerable antioxidant potential, as demonstrated by DPPH and ABTS radical scavenging assays. These findings were supported by FRAP and CUPRAC assays, which confirmed their ferric and cupric ion-reducing capabilities. The stronger antioxidant activity observed for mycelium-derived EVs may be primarily explained by their higher particle concentration compared to fruiting body EVs, as shown by NTA (Figure 1A–D), which likely results in a greater amount of bioactive cargo. Another possible explanation is that EVs from pure-culture mycelium preparations could be less affected by environmental impurities than those obtained from fruiting bodies, leading to higher purity and activity. The higher concentration of bioactive polyphenols in the 40 K fraction contributes to the better antioxidant performance observed, demonstrating a direct link between metabolomic profile and functional activity. These antioxidant effects could be attributed to the bioactive metabolites present within the vesicles, offering potential applications in addressing oxidative stress, as indicated by past research on the antioxidant characteristics of extracellular vesicles derived from mushrooms [46,47]. Nevertheless, while these results provide a solid in vitro indication of antioxidant capacity, further studies using cellular models and in vivo systems are required to validate their functional impact, as well as to assess biodistribution, safety, and therapeutic efficacy.

Based on the characterization and antioxidant activity assays, the 40 K mycelial EVs emerged as the most suitable fraction, displaying superior purity, structural integrity, and bioactivity. The superior quality of 40 K fraction can be related to the enriched metabolomic content, including polyphenols, amino acids, and nucleosides, which together contribute to the observed bioactivity.

Together, these findings highlight the functional and biochemical diversity of EVs obtained from *P. eryngii* mycelium, emphasizing their potential role in creating natural bioactive products. Based on their metabolite composition, such bioactive products may include antioxidant and anti-inflammatory nutraceutical formulations enriched in polyphenols (e.g., (−)-epicatechin, caffeic acid, gallic acid), as well as functional food supplements aimed at supporting redox balance and metabolic health. In addition, the presence of sphingolipids and phosphatidylcholines suggests possible applications of these EVs in nanocarrier-based delivery systems for therapeutic purposes. Additionally, the mycelium’s advantage over the fruiting body in terms of EV yield, as well the 40 K fraction advantage over the 100 K in terms of purity underscores important implications for the use of MDEVs. Moreover, the integration of untargeted metabolomic data provides mechanistic insight into how MDEVs may deliver multifaceted biological effects, supporting their development as functional nanoparticles in biotechnology or nutraceutical applications.

## 4. Materials and Methods

### 4.1. Mushroom Material

Fruiting bodies of *Pleurotus eryngii* were collected on Polvese Island (Perugia, Italy) and identified based on both morphological and molecular analyses, as described by Angeles Flores [15]. Voucher specimens were deposited in the PERU herbarium at the Department of Chemistry, Biology and Biotechnology, University of Perugia. For mycelial isolation, small tissue fragments (5 × 5 × 5 mm) were aseptically excised from the context of fresh basidiomata and transferred to Petri dishes containing Rose Bengal Chloramphenicol Agar. Plates inoculated with 3–4 explants were incubated in the dark at 25 °C for 7 days. Once pure mycelium was obtained, it was transferred to Petri dishes containing Malt Extract Agar (MEA) and incubated at 25 °C until the mycelium fully colonized the medium. Subsequently, a small fragment of the mycelium was transferred to Erlenmeyer flasks containing 25 mL of sterile ME liquid medium. The cultures were incubated on a rotary shaker at 180 rpm at 25 °C until complete mycelial growth was achieved, following the protocol described in Ref. [26].

### 4.2. Purification of Mushroom-Derived Extracellular Vesicles and P. eryngii Crude Extract

**Figure 6:**
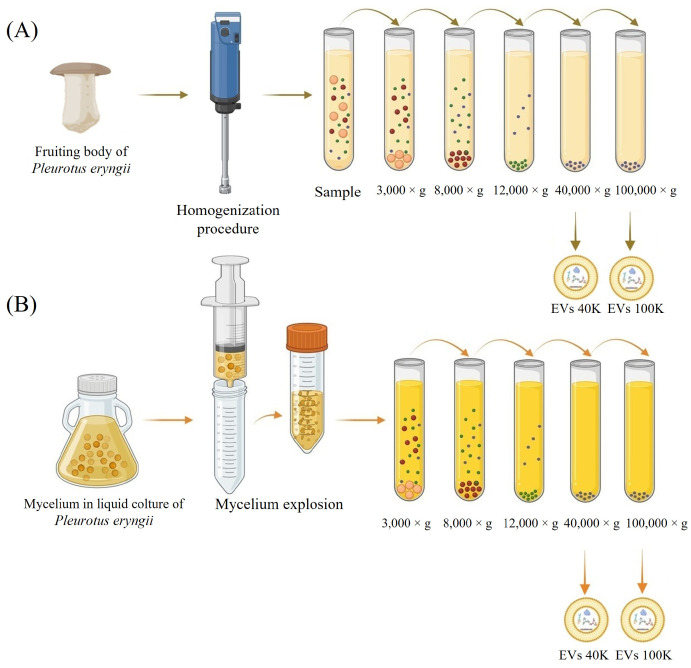
Schematic representation of the extracellular vesicle (EV) purification workflow. (**A**) EVs isolation protocol for *P. eryngii* fruiting body; (**B**) EVs isolation protocol for *P. eryngii* liquid-cultured mycelium.

Extracellular vesicles (EVs) were purified from both the fruiting body and the mycelium of *Pleurotus eryngii*. For the fruiting body, 5 g of fresh tissue (including stem and cap) were homogenized in cold phosphate-buffered saline (PBS) for 30 s using an IKA T25 Ultra-Turrax (IKA, Breisgau, Germany). For the mycelium, liquid-cultured biomass was transferred into a 50 mL tube and mechanically fragmented by passing it through a 50 mL syringe. Both homogenates were subjected to sequential low-speed centrifugation steps to remove cells, cell debris, and large vesicles (3000× *g* for 15 min, 8000× *g* for 20 min, and 12,000× *g* for 30 min at 4 °C). The resulting supernatant was ultracentrifuged at 40,000× *g* for 70 min at 4 °C. The obtained pellet was washed with PBS and ultracentrifuged again under the same conditions to yield the 40 K fraction. To obtain the 100 K fraction, the supernatant was subsequently ultracentrifuged at 100,000× *g* for 70 min at 4 °C. The purification procedure is schematically summarized in Figure 6.

For the preparation of *P. eryngii* crude extract, 2 g of fresh material was homogenized with 10 mL of ethanol (70%) and left overnight at 4 °C under stirring. Each sample was then centrifuged at 15,000× *g* for 10 min at 4 °C.

### 4.3. Characterization of Mushroom-Derived Extracellular Vesicles

#### 4.3.1. Scanning Electron Microscopy (SEM) and Nanoparticle Tracking Analysis (NTA)

For scanning electron microscopy (SEM), EVs were resuspended in 100 µL of PBS Buffer (0.22 µm filtered) and the protein concentration was quantified by Bradford method [48]. 3 µg of proteins were collected, diluted in 2 mL of 2.5% glutaraldehyde (*v*/*v* in 1X PBS), and fixed for 15 min at room temperature, as reported in previous studied [49,50]. Subsequently, the samples were diluted in 15 mL of deionized water (filtered through 0.22 µm filters) and washed twice using Vivaspin tubes (300 kDa cut-off) (Sartorius, Göttingen, Germany), followed by centrifugation at 3000× *g* for 3 min. Upon removal of the eluate, extracellular vesicles were washed twice with 10 mL of water and centrifuged under the same conditions. A single dilution (1:2000) was prepared, and 20 µL were applied onto 12 mm glass coverslips. Samples were then fixed and coated with a thin conductive layer prior to electron microscopy imaging. SEM images were obtained using a field emission gun electron scanning microscope (LEO 1525 Zeiss; Thornwood, NY, USA) after Cr metallization using a high-resolution sputter Q150T ES-Quorum apparatus (24 s sputter at a current of 240 mA, Electron Microscopy Sciences, Hatfield, PA, USA). Chromium thickness was ~10 nm [51].

A NanoSight NS300 instrument (Malvern, Westborough, MA, USA) was used to assess the size distribution and concentration of *P. eryngii* EVs. EVs were resuspended and diluted in filtered PBS (filtered through a 0.22 μm filter). For each sample, five 60 s videos were captured, processed using NTA 2.3 software.

#### 4.3.2. Immunoblotting Analysis

To investigate both the overall protein profile and the presence of the vesicular marker CD81 in MDEVs from *P. eryngii*, samples obtained by ultracentrifugation at 40,000× *g* or 100,000× *g*, as well as those isolated via density gradient centrifugation, were resuspended in 50 μL of PBS. CD81, a standard marker for animal EVs, was selected as a comparative marker in line with previous fungal EV studies, acknowledging potential evolutionary divergence. Aliquots containing 10 μg of total protein were mixed with 5× sample buffer (1 M Tris-HCl pH 6.8, 5% SDS, 6% glycerol, 0.01% bromophenol blue) supplemented with 125 mM DTT. Samples were heated at 95 °C for 5 min and subjected to SDS-PAGE on 10% acrylamide gels. Gels were either stained with Coomassie Brilliant Blue G-250 to assess the protein composition or transferred onto polyvinylidene difluoride (PVDF) membranes using the Trans-Blot Turbo Transfer System (Bio-Rad, Hercules, CA, USA). After blocking, membranes were incubated overnight at 4 °C with a primary antibody against CD81 (Santa Cruz Biotechnology, Dallas, TX, USA). Detection was performed using HRP-conjugated secondary antibodies (Cell Signaling Technology, Beverly, MA, USA) and visualized via enhanced chemiluminescence (ECL) (GE Biosciences, Piscataway, NJ, USA).

### 4.4. Scavenging Activity

The antioxidant activity of MDEVs isolated from the fruiting body and mycelium of *P. eryngii* (fractions 40 K and 100 K) was evaluated using four complementary in vitro assays: DPPH, ABTS, FRAP and CUPRAC. All measurements were performed in 96-well microplates, and absorbance was recorded with an Infinite^®^ Tecan plate reader (Tecan Group Ltd., Männedorf, Switzerland). Standard curves were generated for each assay, and Trolox was used as a reference control in all cases.

In the DPPH (2,2-diphenyl-1-picrylhydrazyl) assay, 50 µL of EV sample were added to 150 µL of a 0.1 mM DPPH ethanolic solution. The mixture was incubated in the dark for 30 min at room temperature, and absorbance was read at 517 nm [52]. For the ABTS assay, the ABTS^+^ radical was generated by reacting 7 mM ABTS with 2.45 mM potassium persulfate and leaving the solution in the dark for 12–16 h. The solution was then diluted in ethanol to obtain an absorbance of 0.70 ± 0.02 at 734 nm. In each well, 150 µL of the ABTS^+^ solution were mixed with 50 µL of EV sample, and absorbance was measured at 734 nm after 6 min of incubation at room temperature in the dark [53].

The scavenging activity for both assays was calculated using the following equation:

$$\%Scavenging=\left(\frac{A_{0}-A_{1}}{A_{0}}\right)*100$$

where **A_0_** is the absorbance of the control (reagent + solvent) and **A_1_** is the absorbance in the presence of the sample. Results were expressed as % inhibition.

Ferric reducing antioxidant power (FRAP) was assessed by mixing 10 µL of EVs with 190 µL of FRAP reagent, prepared by combining 300 mM acetate buffer (pH 3.6), 10 mM TPTZ in 40 mM HCl, and 20 mM FeCl_3_ in a 10:1:1 volume ratio. The reaction was incubated in the dark for 30 min at room temperature, and absorbance was measured at 593 nm [52].

Cupric ion reducing antioxidant capacity (CUPRAC) was determined by mixing 30 µL of EV sample with 170 µL of CUPRAC reagent, composed of 10 mM Cu(II), 7.5 mM alcoholic neocuproine solution, and 1 M ammonium acetate buffer (pH 7) in a 1:1:1 volume ratio. After incubation for 30 min in the dark, absorbance was measured at 450 nm. Results, for all the assays, were normalized to EV protein content (determined by Bradford assay) and were expressed as micrograms of Trolox equivalents per microgram of EVs. All assays were conducted in triplicate [52].

### 4.5. Determination of Total Phenolic Content (TPC)

Folin–Ciocalteu colorimetric method was used to determine the total phenolic content of 40 K mycelium MDEVs, selected based on their superior performance in previous antioxidant assays. A standard calibration curve was generated using gallic acid as the reference compound. For the assay, performed following previously described protocols with minor modifications [54], 50 µL of 40 K mycelium MDEVs were mixed with 150 µL of Folin–Ciocalteu reagent, previously diluted 1:10 with deionized water. The mixture was vortexed and incubated for 5 min at room temperature. Subsequently, 150 µL of a 20% (*w*/*v*) sodium carbonate (Na_2_CO_3_) solution was added. The reaction was carried out in the dark for 30 min at room temperature, and absorbance was measured at 750 nm using an Infinite^®^ Tecan spectrophotometer (Tecan Group Ltd., Männedorf, Switzerland). Results were expressed as micrograms of gallic acid equivalents (µg GAE) per µg of MDEVs.

### 4.6. Untargeted LC-MS/MS-Based Metabolomics Analysis of MDEVs

Untargeted metabolomic profiling of 40 K mycelium MDEVs was performed to investigate both polar and apolar compounds. EVs were previously isolated by ultracentrifugation and stored at −80 °C until extraction.

For lipid extraction (apolar fraction), a modified MMC protocol was applied as previously described by Pellegrino et al. [37]. Briefly, 50 µL of EV suspension was mixed with 1 mL of a methanol/methyl tert-butyl ether/chloroform mixture (1:1:1, *v/v/v*). After vortexing, samples were centrifuged at 13,000× *g* for 10 min at 4 °C. The supernatant was collected, evaporated with flow of nitrogen at 60 °C, and reconstituted in methanol/toluene (9:1 *v*/*v*) for the LC/MS analysis.

Polar metabolites were extracted following the method reported by Cajka et al. [55] with minor modifications. EVs samples were mixed with 275 µL of cold methanol and vortexed. Then 1 mL of MTBE was added to each sample, vortexed and shaken with thermomixer at 22 °C for 20 min. 275 µL of MeOH 10% solution was added to the samples to separate the phases. Subsequently, the samples were centrifuged at 13,000× *g* for 10 min at 4 °C. The lower aqueous phase (polar fraction) was recovered, dried under a steam of Nitrogen and reconstituted with 100 µL of ACN/H_2_O (4:1 *v*/*v*) for the LC/MS analysis.

As part of the metabolomics analysis, the presence of polyphenolic compounds was also investigated using an untargeted LC-QTOF approach. The polyphenols of the EVs were extracted using an extraction solution consisting of 70% methanol containing 3% formic acid (40 µL) and centrifuged after vortexing. The supernatant was recovered for the LC-MS analysis.

All samples were analyzed by LC-QTOF using an Agilent 1260 Infinity II liquid chromatography System coupled to an Agilent 6530 Q-TOF spectrometer (Agilent Technologies, Santa Clara, CA, USA).

Chromatographic separation of lipids was performed using a Waters C18 Acquity UPLC column (100 × 2.1 mm, particle diameter: 1.7 µm) (Waters, Milford, MA, USA) maintained at 60 °C with a flow rate of 0.30 mL/min. The mobile phase consisted of two eluents: A: (ACN 60% + H_2_O 40%) + 10 mM Ammonium Acetate, and B: (IPA 90% + ACN 10%) + 10 mM Ammonium Acetate (check Table S2 for the gradient). For the polyphenols, we used the same column with different solvents (eluent A: H_2_O + 0.2% Formic acid, and eluent B: ACN 0.2% Formic acid) (check Table S3 for the gradient).

For the polar metabolites, chromatographic separation was performed on a Waters XBridge BEH Amide column (dimensions: 150 × 2.1 mm, particle diameter: 2.5 µm) at 25 °C. The mobile phase consisted of two eluents: Water (A), and Acetonitrile 95% (B) both with 0.125% formic acid and 10 ammonium formate. For the gradient check Table S4.

Mass spectrometric acquisition was performed in both positive and negative electrospray ionization (ESI) modes over an m/z range of 40–1700, using the Agilent JetStream source (Agilent Technologies, Santa Clara, CA, USA).

Raw data were processed using MS-DIAL software (v4.48) for peak detection, alignment, integration, and annotation [56]. A data matrix was generated including the accurate mass and peak area for each detected feature. Nist2020 database was used for the annotation of polar metabolites and polyphenols. For lipid annotation, it has been used LSG [57] to generate the databases of lipids.

The open-source tool LipidONE 2.3 [58] was used to analyze the lipidomic profile, with five biological replicates per treatment group. Statistical analysis was performed using a *t*-test.

## 5. Conclusions

This study contributes to the growing interest in fungal extracellular vesicles by shedding light on the characteristics and antioxidant potential of *P. eryngii*-derived EVs. The findings highlight the relevance of mycelium-derived vesicles, particularly from the 40 K fraction, as promising candidates for further investigation in biological environment.

Although much remains to be understood, these results lay the groundwork for future studies aimed at exploring the biological properties of EVs derived from edible fungi. Particular attention should be given to their effects on cell systems, including potential antioxidant, cytotoxic, and anti-inflammatory activities. Such investigations will be crucial to clarify the functional role of fungal vesicles and to assess their value in biomedical and biotechnological applications. Nevertheless, this is preliminary in vitro study, and therefore its findings cannot be directly extrapolated to complex biological systems. For these reasons, some important biological effects, such as immunomodulatory or anti-inflammatory effects, must be explored. At present, the therapeutic use of mycelium-derived vesicles remains a perspective that requires extensive experimental validation. While their bioactive properties suggest a promising role in the development of innovative, nature-inspired strategies for health and therapeutic innovation, such applications should be considered as potential promising outcomes.

## Figures and Tables

**Table 1:** Polyphenol content in *P. eryngii*-derived EVs and *P.eryngii* extract (mean ± SE, *n* = 5). Statistical analysis was performed using *t*-test. Asterisks indicate significant differences in relative content between EVs and extract: ** *p* < 0.01, *** *p* < 0.001, ns = not significant.

Polyphenols	*P. eryngii* Mycelium-Derived EVs	*P. eryngii* Extract	*p*-Value
Caffeic acid	6.290 ± 0.126	1.269 ± 0.115	***
epicatechin gallate	22.352 ± 0.303	32.198 ± 2.153	**
(−)-epicatechin	41.141 ± 0.461	27.256 ± 4.569	**
Rutin	6.389 ± 0.075	3.677 ± 0.093	**
Chlorogenic acid	2.041 ± 0.034	2.031 ± 0.011	ns
Gallic acid	1.307 ± 0.032	0.251 ± 0.037	***
Salicylic acid	7.312 ± 0.077	31.874 ± 0.678	***
trans-4-Coumaric acid	13.166 ± 0.107	1.441 ± 0.144	***

**Table 2:** Antioxidant capacity of MDEVs derived from mycelium and fruiting body.

	40 K		*p*	100 K		*p*
	FB	Myc		FB	Myc	
DPPH (µg TE/µg EVs)	25.96 ± 1.27	56.27 ± 0.48	****	17.68 ± 2.75	64.73 ± 1.84	****
ABTS (µg TE/µg EVs)	14.18 ± 1.51	89.61 ± 0.32	****	11.17 ± 0.18	117.15 ± 0.66	****
FRAP (µg TE/µg EVs)	15.92 ± 1.12	113.34 ± 5.04	****	17.99 ± 5.86	64.32 ± 1.77	***
CUPRAC (µg TE/µg EVs)	14.72 ± 0.83	196.5 ± 2.76	****	9.01 ± 0.36	76.73 ± 7.38	****

DPPH 2,2-diphenyl-1-picrylhydrazyl, ABTS 2,2′-azino-bis(3-ethylbenzothiazoline-6-sulfonic acid), CUPRAC cupric ion reducing antioxidant capacity, FRAP ferric reducing antioxidant power, FB Fruiting body, Myc Mycelium. Values are reported as mean ± standard deviation (*n* = 3). Statistical analysis was performed using the *t*-test. **** *p* < 0.0001; *** *p* < 0.001.

## Data Availability

Data are provided within the manuscript.

## Supplementary Materials

The following supporting information can be downloaded at: https://www.mdpi.com/article/10.3390/ph18091362/s1, Table S1. Identified Metabolites in 40 K and 100 K mycelium MDEVs. Table S2. Gradient table for liquid chromatographic separation of lipids. Table S3. Gradient table for liquid chromatographic separation of polyphenols. Table S4. Gradient table for liquid chromatographic separation of polar metabolites. Figure S1. Semi-quantitative analysis of different molecular species detected in Ceramide (Cer) lipid class (A), phosphatidylcholine (PC) lipid class (B), Phosphatidylethanolamines (PE) lipid class (C), phosphatidylserines (PS) lipid class (D) and sphingomyelin (SM) (E). Data are expressed as mean ± ES (*n* = 3). Statistically significant differences between fractions are indicated as * *p* < 0.05.

## Abbreviations

The following abbreviations are used in this manuscript:

ABTS	2,2′-Azino-bis(3-ethylbenzothiazoline-6-sulfonic acid)
ACN	Acetonitrile
Cer	Ceramide
CUPRAC	Cupric Ion Reducing Antioxidant Capacity
DG	Diacylglycerol
DPPH	2,2-Diphenyl-1-picrylhydrazyl
ECL	Enhanced Chemiluminescence
EVs	Extracellular Vesicles
FB	Fruiting Body
FRAP	Ferric Reducing Antioxidant Power
GAE	Gallic Acid Equivalent
IPA	Isopropanol
LC-MS/MS	Liquid Chromatography–Tandem Mass Spectrometry
MDEVs	Mushroom Derived Extracellular Vesicles
ME	Malt Extract
MTBE	Methyl tert-butyl ether
Myc	Mycelium
NTA	Nano-Particle Tracking Analysis
PBS	Phosphate-Buffered Saline
PC	Phosphatidylcholine
PE	Phosphatidylethanolamine
PS	Phosphatidylserine
PVDF	Polyvinylidene Difluoride
QTOF	Quadrupole Time-of-Flight
SD	Standard Deviation
SE	Standard Error
SEM	Scanning Electron Microscopy
TE	Trolox Equivalent
TG	Triacylglycerol
TPC	Total Phenolic Content
TPTZ	2,4,6-Tripyridyl-s-triazine
UPLC	Ultra-Performance Liquid Chromatography
