# Supplementary material for: Therapeutic Potential of Bioactive Compounds in Edible Mushroom-Derived Extracellular Vesicles: Isolation and Characterization of EVs from Pleurotus eryngii
Source: Pharmaceuticals (Basel). 2025 Sep 12;18(9):1362. doi: 10.3390/ph18091362 (PMC12472452; doi:10.3390/ph18091362)
Supplement: Supplementary file 1 [file pharmaceuticals-18-01362-s001.zip › pharmaceuticals-3844968-supplementary.pdf]

Table S1. Identified Metabolites in 40K and 100K mycelium MDEVs.

**Class**	**Compound Name**	**% Relative abundance**	
		40K	*100K*
Alkaloids	Harmane	0.644 ± 0.116	0.517 ± 0.114
	Norharmane	0.642 ± 0.230	0.856 ± 0.215
	Quinaldic acid	0.079 ± 0.018	0.057 ± 0.002
	Quinolin-6-ol	0.025 ± 0.011	0.023 ± 0.001
Amine	Phenylethanolamine	2.860 ± 0.459	2.694 ± 0.155
Amino acids and peptides	γ-Aminobutyric acid	0.076 ± 0.031	0.127 ± 0.007
	γ-L-Glutamyl-L-alanine	3.606 ± 0.768	2.989 ± 0.167
	1,2-Diamino-2-methylpropane	1.569 ± 0.177	1.493 ± 0.032
	Cyclo(isoleucylprolyl)	0.868 ± 0.153	0.432 ± 0.020
	Glu-Pro	0.049 ± 0.023	0.051 ± 0.005
	Glycyl-L-4-hydroxyproline	0.062 ± 0.054	0.109 ± 0.006
	L-Arginine	3.496 ± 0.808	4.208 ± 0.264
	L-Asparagine	0.118 ± 0.028	0.126 ± 0.009
	L-Glutamic acid	0.760 ± 0.180	0.798 ± 0.049
	L-Glutamine	0.260 ± 0.067	0.238 ± 0.017

	L-Histidine	3.544 ± 0.414	3.404 ± 0.164
	L-Isoleucine	5.720 ± 0.973	5.740 ± 0.205
	L-Leucine	9.094 ± 1.014	8.660 ± 0.486
	L-Lysine	2.995 ± 0.395	2.845 ± 0.142
	L-Phenylalanine	7.888 ± 1.101	7.405 ± 0.402
	L-Tryptophan	0.318 ± 0.188	0.453 ± 0.035
	L-Tyrosine	0.180 ± 0.078	0.246 ± 0.001
	L-Valine	3.597 ± 0.394	3.446 ± 0.070
	N-.alpha.-(tert-Butoxycarbonyl)-L-histidine	0.473 ± 0.057	0.456 ± 0.022
	N-Acetyl-L-alanine	0.146 ± 0.043	0.166 ± 0.004
	NG,NG-Dimethyl-L-arginine	0.368 ± 0.050	0.386 ± 0.033
	N-L-.γ-Glutamyl-L-leucine	7.199 ± 1.080	5.573 ± 0.922
	Phe-Pro	1.005 ± 0.524	1.032 ± 0.113
	Pro-Val	0.327 ± 0.198	0.366 ± 0.013
	PyroGlu-Val	1.711 ± 0.245	1.396 ± 0.041
Choline derivatives	Betaine	19.349 ± 3.915	20.518 ± 0.675
	Choline	7.761 ± 1.815	8.536 ± 0.405
	Glycerophosphocholine	1.224 ± 0.382	1.335 ± 0.040

Indole derivatives	3-(2-Hydroxyethyl)indole	0.544 ± 0.340	0.678 ± 0.044
	3-Hydroxy-3-methyl-2,3-dihydro-1H-indol-2-one	0.463 ± 0.074	0.331 ± 0.013
Imidazole derivatives	1-(2-Carboxyethyl)-1H-imidazole	0.009 ± 0.001	0.009 ± 0.000
Organic acids	3-Hydroxy-3-methylglutaric acid	0.685 ± 0.155	0.700 ± 0.028
	Allantoic acid	0.012 ± 0.002	0.011 ± 0.001
	Citric acid	0.075 ± 0.032	0.013 ± 0.009
	Isocinchomeronic acid	0.095 ± 0.020	0.108 ± 0.005
	Succinic acid	1.237 ± 0.477	1.406 ± 0.050
	Sulfoacetic acid	0.005 ± 0.003	0.007 ± 0.001
Nucleosides and nucleobases	Adenosine	1.618 ± 0.670	2.063 ± 0.147
	Guanosine	0.732 ± 0.319	0.868 ± 0.050
	Hypoxanthine	2.365 ± 0.752	2.421 ± 0.204
	Inosine	0.694 ± 0.233	0.726 ± 0.068
Sugars	D-(+)-Trehalose	0.451 ± 0.097	0.402 ± 0.053
	D-Fructose	0.360 ± 0.167	0.592 ± 0.029
	D-Lyxose	0.260 ± 0.071	0.268 ± 0.011

	Sucrose	0.479 ± 0.082	0.509 ± 0.038
Vitamins and cofactors	Geranyl pyrophosphate	0.008 ± 0.001	0.008 ± 0.000
	L-Carnitine	0.300 ± 0.138	0.418 ± 0.030
	Nicotinic acid	0.901 ± 0.169	0.993 ± 0.076
	Pantothenic acid	0.428 ± 0.115	0.454 ± 0.025

* Results are reported as % relative abundance that indicates the proportion of each metabolite's peak area relative to the sum of all detected metabolites in the respective EV fraction.

Figure S1. Semi-quantitative analysis of different molecular species detected in Ceramide (Cer) lipid class (A), phosphatidylcholine (PC) lipid class (B), Phosphatidylethanolamines (PE) lipid class (C), phosphatidylserines (PS) lipid class (D) and sphingomyelin (SM) (E). Data are expressed as mean ± ES (n = 3). Statistically significant differences between fractions are indicated as *p < 0.05.

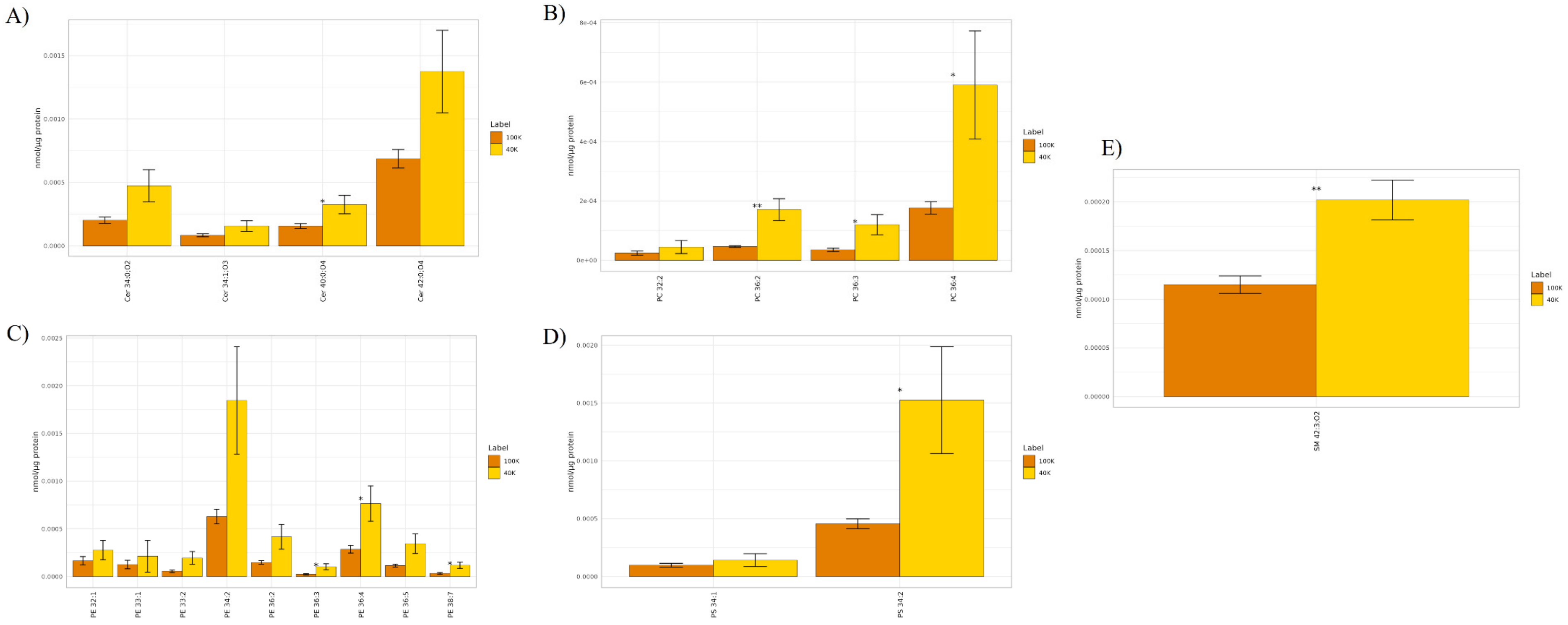

Table S2. Gradient table for liquid chromatographic separation of lipids

Time (min)	Eluent A (%)	Eluent B (%)
**0**	60	40
**1**	45	55
**13**	5	95
**14**	5	95
**16.2**	60	4
**20**	Stop	Stop

Eluent A: (ACN 60% + H2O 40%) + 10mM Ammonium Acetate;

Eluent B: (IPA 90% + ACN 10%) + 10mM Ammonium Acetate

Table S3. Gradient table for liquid chromatographic separation of polyphenols

Time (min)	Eluent A (%)	Eluent B (%)
**0**	95	5
**15**	55	45
**18**	5	95
**20**	5	95
**20.1**	95	5
**23**	Stop	Stop

Eluent A: H2O + 0.2% Formic acid; Eluent B: ACN 0.2% Formic acid

Table S4. Gradient table for liquid chromatographic separation of polar metabolites

Time (min)	Eluent A (%)	Eluent B (%)
**0**	0	100
**0.5**	0	100
**8**	50	50
**8.1**	0	100
**9.5**	0	100
**13.5**	Stop	Stop

Eluent A: H2O + 0.125% Formic acid + 10 Ammonium Formate; Eluent B: (ACN 95% + H2O 5%) + 0.125% Formic acid + 10 Ammonium Formate
